# Noninvasive remote activation of the ventral midbrain by transcranial direct current stimulation of prefrontal cortex

**DOI:** 10.1038/tp.2013.44

**Published:** 2013-06-11

**Authors:** V S Chib, K Yun, H Takahashi, S Shimojo

**Affiliations:** 1 Division of Biology, California Institute of Technology, Pasadena, CA, USA; 2 Computation and Neural Systems, California Institute of Technology, Pasadena, CA, USA; 3 Department of Psychiatry, Kyoto University Graduate School of Medicine, Kyoto, Japan; 4 Precursory Research for Embryonic Science and Technology (PRESTO), Japan Science and Technology Agency, Kawaguchi, Japan

**Keywords:** brain stimulation, midbrain, preference, prefrontal cortex, transcranial direct current stimulation (tDCS)

## Abstract

The midbrain lies deep within the brain and has an important role in reward, motivation, movement and the pathophysiology of various neuropsychiatric disorders such as Parkinson's disease, schizophrenia, depression and addiction. To date, the primary means of acting on this region has been with pharmacological interventions or implanted electrodes. Here we introduce a new noninvasive brain stimulation technique that exploits the highly interconnected nature of the midbrain and prefrontal cortex to stimulate deep brain regions. Using transcranial direct current stimulation (tDCS) of the prefrontal cortex, we were able to remotely activate the interconnected midbrain and cause increases in participants' appraisals of facial attractiveness. Participants with more enhanced prefrontal/midbrain connectivity following stimulation exhibited greater increases in attractiveness ratings. These results illustrate that noninvasive direct stimulation of prefrontal cortex can induce neural activity in the distally connected midbrain, which directly effects behavior. Furthermore, these results suggest that this tDCS protocol could provide a promising approach to modulate midbrain functions that are disrupted in neuropsychiatric disorders.

## Introduction

Dopaminergic neurons in the substantia nigra (SN) and ventral tegmental area (VTA) project to numerous areas in the brain.^1, 2, 3, 4, 5, 6^ Impairments in dopaminergic function of the SN/VTA (which we will collectively refer to as the ventral midbrain) have been implicated in the pathophysiology of a number of neurological and neuropsychiatric disorders such as Parkinson's disease, depression and addiction.^7, 8, 9, 10^ Given that SN/VTA neurons lie deep within the brain, the primary means of influencing them have been with systematic pharmacological interventions^11^ or implantation of deep brain stimulators.^12^ Systematic pharmacological intervention is the first line of therapy for many neurological and neuropsychiatric disorders;^13^ however, it precludes from region-specific interventions, and some portions of patients with these disorders do not respond to the drug therapy. For such non-responders, invasive deep brain stimulation may be considered.^14^

The two predominant means of non-invasively stimulating the brain are transcranial magnetic stimulation (TMS) and transcranial direct current stimulation (tDCS).^15^ TMS involves inducing an electrical current within the brain via a pulsating magnetic field applied by an induction coil stimulator placed above the scalp. tDCS involves the application of a small current between anodal and cathodal electrodes placed on the scalp. Both of these stimulation methods result in changes in brain function by causing neurons' resting membrane potential to depolarize or hyperpolarize. Positive stimulation (as in the cases of TMS and anodal tDCS) causes a depolarization of the resting membrane potential, leading to increases in neuronal excitability and more spontaneous cell firing. Negative stimulation (in the cases of cathodal tDCS) causes hyperpolarization of the resting membrane potential, leading to decreases in neuronal excitability and decreased spontaneous cell firing. tDCS is generally applied in order to induce cortical changes that persist after stimulation, while TMS can be used to induce online cortical changes as well as changes that persist after stimulation. In both the cases, the duration and effects of stimulation increase as the duration of stimulation increases and the strength of the current increases. The benefits of tDCS over TMS, from a logistic therapeutic perspective, are that tDCS units are extremely inexpensive and easily mobile in comparison to TMS stimulators. For a thorough overview of tDCS, see Nitsche *et al.*^16^

tDCS and TMS have been used to probe neurocircuitry and treat neurological disorders in numerous studies.^16, 17, 18, 19, 20, 21, 22, 23^ Given that TMS and tDCS are only capable of stimulating the cortical surface, these studies have mainly focused on direct stimulation of a cortical region below the stimulation electrodes, rather than exploiting the interconnected neural network to induce remote changes in deep brain activity. It should be noted that two non-invasive brain stimulation studies reported cortical stimulation-induced activations in the caudate nucleus;^24, 25^ however, they are difficult to interpret as neither revealed functional connectivity that was directly related to stimulation-induced behavioral changes. More recently, a repetitive TMS study found that stimulation of motor cortex could induce changes in activity in the caudate and that stimulation-induced changes in connectivity between these areas were related to behavioral performance during a cognitive switching task.^26^

One recent study examining the effectiveness of anodal tDCS of frontal cortex in rodents found significantly increased neural activity in the frontal cortex and interconnected midbrain regions following stimulation.^27^ Critically, the authors also found an increase in intracellular dopamine in these distal regions. They suggested that the increases in activity and intracellular dopamine in the midbrain were caused by the direct tDCS of the frontal cortex. This study alludes to the exciting possibility of exploiting the highly interconnected nature of cortical brain regions to stimulate deep brain dopaminergic areas that are not directly accessible with noninvasive stimulation methods. The results from this rodent study inspired us to test if such increased midbrain dopaminergic functions, in response to anodal tDCS of frontal cortex, could be induced using a similar paradigm in humans.

Given the superficial nature (that is, close to the cortical surface) of the prefrontal cortex, as well as its direct^28, 29, 30, 31, 32^ and indirect efferent projections (via the striatum)^5, 33, 34, 35^ and functional synchrony^36, 37, 38^ with the ventral midbrain, the ventromedial prefrontal cortex (VMPFC) and dorsolateral prefrontal cortex (DLPFC) struck us as excellent locations to directly stimulate to yield remote deep brain activity in humans. A number of studies have associated increases in VMPFC activity^39, 40, 41^ and decreases in DLPFC activity,^42, 43, 44^ with increases in midbrain activity and intracellular dopamine. Furthermore, DLPFC has been implicated in monitoring goal-directed behaviors and valuations that are encoded by the VMPFC.^45, 46, 47^ With these relationships in mind, we hypothesized that exitatory/anodal and inhibitory/cathodal tDCS electrode placement over VMPFC and DLPFC, respectively, would result in the remote activation of the ventral midbrain. Moreover, we reasoned that cathodal stimulation of DLPFC would suppress its control over VMPFC, which would boost the anodal stimulation of the latter, and this enhanced VMPFC stimulation would yield an increased remote activation of the distally interconnected ventral midbrain. We further hypothesized that this remote activation would manifest behaviorally as increases in participants' rewarding appraisals. We chose this behavioral task because discrimination of facial attractiveness and emotions are commonly disrupted in neuropsychiatric disorders such as depression, schizophrenia and Parkinson's disease).^48, 49, 50, 51^

## Materials and methods

### Participants

Ninety-nine right-handed healthy participants took part in this experiment (mean age, 22.9±3.95 years; range 18–37 years), of which 47 were female. Participants had no history of neurological or psychiatric illness. The California Institute of Technology Review Board approved this study, and all participants gave informed consent.

### Stimuli

Participants made attractiveness ratings of a series of 140 faces generated with computer software (FaceGen; Singular Inversions, Toronto, ON, Canada). Seventy male and 70 female Caucasian/European neutrally expressive faces were randomly constructed. The attractiveness of these faces was rated on an eight-point scale ranging from 0 to 7.

Before the experiment, we had a separate group of observers rate these faces (*n*=20). Based on these ratings, we divided the series of 140 faces into two sets. Using the preliminary ratings, we made sure these two groups of faces were uniformly distributed across the range of attractiveness and had the same means and s.ds. of attractiveness ratings (before-stimulation set: 3.66±1.00; after-stimulation set: 3.66±1.02). These attractiveness-balanced sets of faces allowed us to control for possible mere exposure effects that could occur if the same set of faces were used before and after stimulation.

### Experimental protocol

The experiment was divided into three sessions. During the first session (before stimulation), participants made facial attractiveness judgements. During the second session, participants were stimulated with tDCS for 15 min. Finally, during the final session (after stimulation), participants again made facial attractiveness judgments.

To assess the behavioral effects of tDCS, participants were asked to make attractiveness ratings of faces. As described above, two sets of attractiveness-balanced faces were used. One served as a before-stimulation test set, the other an after-stimulation test set. The faces in each set were presented in randomized order. In every trial, participants were presented with a face to rate the attractiveness on an eight-point scale. Participants made a rating by selecting one of the eight buttons on two button-press response pads. One response pad was placed in each hand of the participant, and ratings progressed from the left hand fourth phalange being 0 to the right hand fourth phalange being 7. Participants had 4 s to make a rating, after which their rating value was presented to them for 1 s, followed by a pseudorandomly (∼1–10 s) jittered blank screen. Trials in which subjects did not make a selection in the allotted time were assigned as ‘missed responses'.

To assess the neural effects of tDCS, the two groups of participants were scanned with functional magnetic resonance imaging (fMRI) while making facial attractiveness ratings. These participants were removed from the scanner during administration of tDCS.

### fMRI data acquisition

Functional imaging was conducted using a 3.0 Tesla Trio MRI scanner to acquire gradient echo T2*-weighted echoplanar (EPI) images with blood oxygenation-level-dependent (BOLD) contrast. To optimize functional sensitivity in the frontal cortex, a key region of interest (ROI), we acquired the images in an oblique orientation of 30° to the anterior commissure–posterior commissure line.^52^ In addition, we used a 12-channel-phased array coil to boost the MRI signal. Each volume of images had 44 axial slices. The imaging parameters were as follows: echo time, 30 ms; field of view, 192 mm^2^; in-plane resolution and slice thickness 3 mm (no gap); repetition time, 2.75 s. Whole-brain high-resolution T1-weighted structural scans (1 × 1 × 1 mm^3^) were acquired for each participant, coregistered with their mean EPI images and averaged across particpiants to permit anatomical localization of the functional activations at the group level.

### tDCS administration

**Figure 1:**
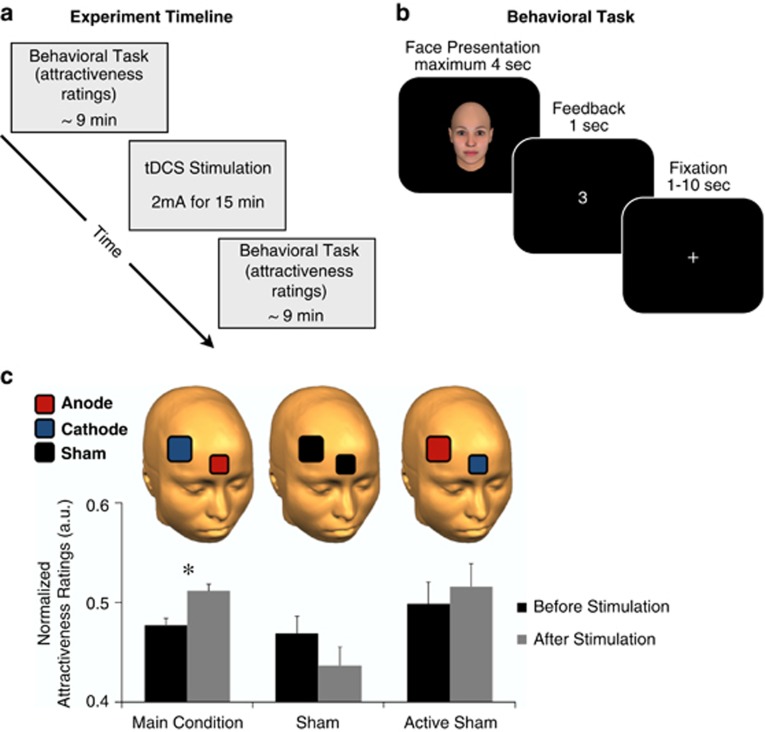
Experimental setup and behavioral results. (**a**) The experiment can be divided into three sessions. During the first session (before stimulation), participants made facial attractiveness judgements. During the second session, participants were stimulated with transcranial direct current stimulation (tDCS) for 15 min. During the final session (after stimulation) participants again made facial attractiveness judgments. The main and active sham groups were scanned with functional magnetic resonance imaging during the before and after stimulation sessions and were removed from the scanner during stimulation. (**b**) At the beginning of each trial of the behavioral task, participants were shown a face and made a rating of how attractive they found the face on a scale from 0–7; 0 being not attractive at all and 7 being very attractive. (**c**) Anodal stimulation of the ventromedial prefrontal cortex (VMPFC) and cathodal stimulation of the right dorsolateral prefrontal cortex (DLPFC) (main stimulation group) resulted in a significant increase in mean attractiveness ratings (*P<0.05). We performed a number of control conditions on separate groups of participants, none of which resulted in a significant increase in attractiveness ratings (For a complete description of the control results, see Supplementary Figure S1). To allow for a more focal stimulation of VMPFC in the main group, we administered tDCS with a small electrode over VMPFC and a large electrode over DLPFC. Error bars denote s.e.m. a.u., arbitrary units.

tDCS was delivered using a battery-driven constant-current stimulator (DC-Stimulator, neuroConn GmbH, Ilmenau, Germany), through conductive-rubber electrodes, placed over two saline-soaked sponges. To allow for more focal stimulation in the main stimulation condition (anode placement over VMPFC, cathode placement over DLPFC), we used two sizes of electrode.^53^ In the main condition, the smaller-sized electrode was 3.5 cm × 3.5 cm (12.25 cm^2^, current density, 0.16 mA cm^−2^) and placed over VMPFC, and the larger-sized electrode was 5 cm × 5 cm (25 cm^2^, current density, 0.08 mA cm^−2^) and placed over DLPFC. Figure 1c and Supplementary Figure S1 show an illustration of the electrode placements in all stimulation conditions.

During active stimulation conditions, tDCS was performed for 15 min and at 2 mA intensity (20 s ramp in and 20 s ramp out). The impedance was controlled by the device, normally ranging <10 kΩ, limited by the voltage at <26 V. Similar stimulation parameters are commonly used to elicit behavioral responses from tDCS.^16^

We localized stimulation sites using a combination of the 10–20 international system for EEG placement and anatomical landmarks. Our experiment involved four stimulation sites of interest (VMPFC, right DLPFC, left DLPFC, vertex). To stimulate the VMPFC, we placed an electrode with its center halfway between Fp1 and Fp2 and over the glabella. To stimulate the right and left DLPFC, we placed an electrode over F4 and F3, respectively. This method of DLPFC localization has been used in tDCS and TMS studies,^54, 55, 56, 57^ and has been confirmed as an accurate method of localization.^58^ To stimulate the vertex, we placed an electrode over the center of Cz. Given tDCS's low spatial resolution and diffuse current spread, it is common to localize stimulation locations using EEG landmarks as opposed to participant-specific neuroanatomy.

We stimulated with six different electrode orientations: Group 1/Main Stimulation Group (*N*=19, 6 females): The anode was placed above the VMPFC and the cathode above the right DLPFC. Group 2/Sham Stimulation Group (*N*=20, 8 females): No stimulation was delivered and electrodes were placed in the same locations as Group 1. Group 3/Active Sham Group (*N*=16, 6 females): The anode was placed above the right DLPFC and the cathode above the VMPFC. Group 4 (*N*=15, 7 females): The anode was placed above the VMPFC and the cathode above the vertex with the center of Cz. Group 5 (*N*=15, 10 females): The anode was placed above the vertex and the cathode above the right DLPFC. Group 6 (*N*=14, 10 females): The anode was placed above the VMPFC and the cathode above the left DLPFC. Participants felt the current as an itching sensation at both electrodes during stimulation. Some participants reported feeling no sensations resulting from stimulation.

We performed these control conditions to confirm that the effects of anodal tDCS of VMPFC and cathodal tDCS of DLPFC (Group 1, main stimulation condition) were specific to this stimulation orientation and polarity. The only condition that resulted in a significant increase in attractiveness ratings was the main stimulation condition (results of all the stimulation groups are shown in Supplementary Figure S1).

We scanned participants in Groups 1 (main stimulation group) and 2 (active sham group) with fMRI during the sessions in which they made attractiveness ratings. We focused our imaging analysis on these two groups to examine the neural effects of the main condition as compared with a control stimulation group that mirrored the main stimulation condition without resulting in a significant behavioral effect. Participants in these groups were removed from the fMRI scanner during the stimulation. This allowed us to examine tDCS-induced changes in neural function associated with significant behavioral changes (main group), as compared with a control stimulation condition that did not result in a significant behavioral effect (active sham group).

### Behavioral data analysis

Raw attractiveness ratings were skewed toward zero. We used max-normalization of the ratings (dividing participants ratings by their maximum attractiveness rating). This normalization allowed us to correct for participants' use of abbreviated ranges of the rating scale. To confirm that the rating data was normal, we performed a Kolmogorov–Smirnov test (before stimulation: *P*=0.163; after stimulation: *P*=0.20).

We used analysis of variance for repeated measures to investigate whether there was a difference between before/after stimulation and the various stimulation groups. Planned comparisons were performed using paired *t*-tests to investigate whether there was a difference between before- and after-stimulation conditions in each group.

### fMRI preprocessing

Image analysis was performed using SPM8 (Wellcome Department of Imaging Neuroscience, Institute of Neurology, London, UK). Images were corrected for slice acquisition time within each volume, motion corrected with realignment to the first volume, spatially normalized to the standard Montreal Neurological Institute EPI template and spatially smoothed using a Gaussian kernel with a full width at half maximum of 8 mm. Intensity normalization and high-pass temporal filtering (using a filter width of 128 s) were also applied to the data.

### fMRI general linear model (GLM)

To analyze the data, we estimated participant-specific (first-level) GLMs using a first-order autoregressive model. This model was designed to identify regions in which BOLD activity was parametrically related to attractiveness ratings and was estimated for the experiment phases in which participants made attractiveness ratings. The GLM included the following regressors for each stimulation condition (before/after stimulation):

1. An indicator function denoting a rating trial, and
2. An indicator function denoting a rating trial multiplied (that is, modulated) by the participants' rating value (0–7 scale) for the face presented in the trial.

Both regressors were modeled as stick functions at the onset of stimulus presentation. The model also included motion parameters, session constants and missed trials as regressors of no interest. The regressors of interest and missed trial regressor were convolved with a canonical form of the hemodynamic response.

This GLM also made use of a parametric regressor. These types of regressors look for areas in which the BOLD response varies with the magnitude of a variable of interest (in this case the attractiveness rating). The estimated coefficient for such regressors can be roughly interpreted as a measure of the strength of association between the BOLD response and the variable of interest.

Single participant contrasts were calculated for the rating parametric regressor separately for the before- and after-stimulation conditions. These contrasts were motivated by previous work and identified regions where BOLD activity is correlated with attractiveness ratings.^59, 60^ We also calculated single participant contrasts for the difference between the parametric regressor for the after-stimulation and before-stimulation conditions. This contrast identifies regions where BOLD activity is more correlated with attractiveness ratings after stimulation than before.

### Group-level analysis

The contrast images computed for each participant were taken to the group random effects level, and conjunctions and comparisons were conducted between Group 1 (main stimulation group) and Group 2 (active sham group) to determine areas showing tDCS-induced changes in activity.

**Figure 2:**
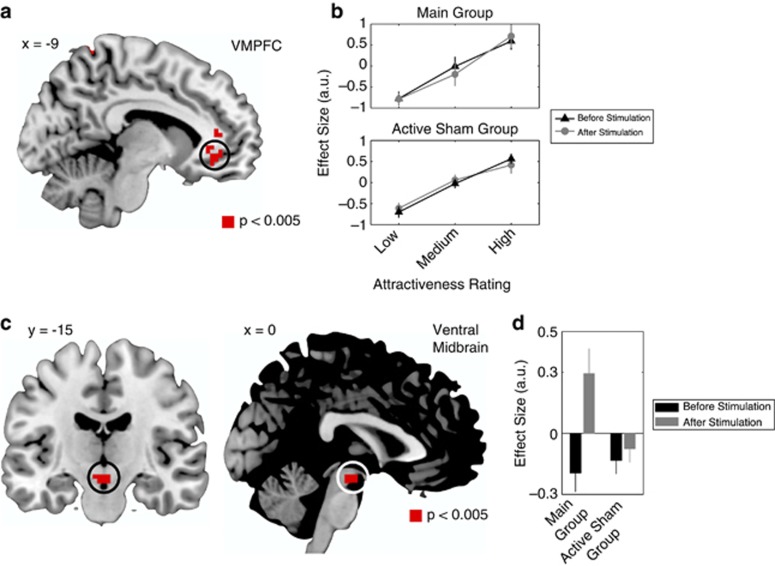
Functional magnetic resonance imaging results. (**a**) A common region of the ventromedial prefrontal cortex (VMPFC) in which activity correlated with attractiveness ratings before and after stimulation, in both the main group and the active sham group. (**b**) The effect size in the VMPFC increased with attractiveness ratings (lower—lower tertile; medium—middle tertile; high—upper tertile). (**c**) An interaction contrast between attractiveness ratings and stimulation revealed a significant increase in ventral midbrain activity in the main group as compared with the active sham group. (**d**) For the interaction contrast, average effect sizes representing the correlation between ventral midbrain activity and rating values before and after stimulation in the main and active sham groups. Ventral midbrain activity was positively correlated with attractiveness ratings after administration of transcranial direct current stimulation. All contrasts are displayed at *P*<0.005 uncorrected, and significant at *P*<0.05, small volume corrected. a.u., arbitrary units.

We computed a conjunction contrast to identify brain areas with overlapping correlations with attractiveness ratings before and after stimulation in both the main and the active sham groups (Figure 2a). We also computed an interaction contrast between attractiveness ratings and before/after stimulation (Figure 2c). For this interaction, we examined differences in activity between the main and active sham groups.

For visualization purposes only, all of the images shown are thresholded at *P*<0.005. For inference purposes, the tables in the Supplementary Information report those areas within *a priori* regions of interest that survive false discovery rate correction. ROI definitions are described below.

### Psychophysiological interactions (PPIs)

The goal of this analysis was to investigate whether anodal tDCS of VMPFC, and simultaneous cathodal tDCS of DLPFC (main stimulation group), caused an increase in the correlation between VMPFC activity and activity in the ventral midbrain compared with the active sham group.

The analysis proceeded in three steps:

First, we computed individual average time series within a 6-mm sphere surrounding individual participant peaks (in both the main and active sham groups) within the functional mask of VMPFC shown in Figure 2a. Variance associated with the six motion regressors was removed from the extracted time series. The location of the peak voxels was based on the GLM described above. The seed time courses were deconvolved, based on the formula for the canonical hemodynamic response, in order to construct a time series of neural activity in the left VMPFC. This was done following the procedures described in Gitelman *et al.*^61^

Second, we estimated a GLM with the following regressors:

1. An interaction between the neural activity in the seed region and an indicator function for before-stimulation and after-stimulation trials,
2. An indicator function for before-stimulation and after-stimulation trials, and
3. The original BOLD eigenvariate (that is, the average time series from the 6-mm sphere.

The first two regressors were convolved with a canonical form of the hemodynamic response function, and the model also included motion parameters as regressors of no interest. The first regressor in this PPI identifies areas that exhibit stimulation-related functional connectivity with VMPFC. In particular, it identifies areas in which the correlation in BOLD activity with VMPFC increases after tDCS. It is important to note that this PPI analysis did not include participants' behavioral ratings and thus revealed neural responses irrespective of the behavioral results.

Third, single participant contrasts for the first regressor were calculated, and a second-level analysis was performed by calculating the main and active sham groups' contrast coefficients.

### *Post-hoc* between-participant regressions

To explore the results further, we performed *post-hoc* linear regressions for the main and active sham groups. We regressed a behavioral measure of the influence tDCS had on attractiveness ratings with a neural measure of the impact tDCS had on connectivity between VMPFC and ventral midbrain, separately for the main and active sham groups. The behavioral measure was calculated by subtracting average ratings of before stimulation from those of after stimulation. The neural measure was the average parameter estimate extracted from the anatomical ROI in the ventral midbrain from the PPI, separately for each stimulation group (main and active sham).

### fMRI ROI analysis

All results reported in the main text are with a corrected significance threshold of *P*<0.05 based on a small-volume false discovery rate correction within the predefined ROIs.

Evaluating the precise location of midbrain fMRI signals is difficult given the small size of the dopaminergic nuclei and problems with group registration in this region.^65^ Therefore, we anatomically defined an ROI for the ventral midbrain (encompassing both the VTA and the SN; Supplementary Figure S2). We also used an anatomically defined ROI for the caudate (Supplementary Figure S2). For our ROI of VMPFC, we defined a 10-mm sphere centered at (x=−3; y=38; z=−18). These coordinates were taken from a previous study examining facial attractiveness encoding.^60^

All effect sizes within these ROIs were extracted using the average of all voxels within the ROI.

## Results

To test our hypotheses, we stimulated participants with tDCS, before and after we had them make attractiveness ratings of a series of faces while being scanned with fMRI (Figure 1a). This procedure allowed us to examine the neural and behavioral influence of our tDCS paradigm on appraisal of facial attractiveness (Figure 1b). We chose this task, because it is known to recruit components of neural reward circuits.^59, 60, 62, 63, 64^ Rating facial attractiveness is one of the most basic reward appraisal tasks and employs limited cortical regions of the prefrontal cortex (that is, orbitofrontal cortex and VMPFC), which allows for a more straightforward interpretation of our behavioral and neural results and fewer confounds of electrode placement.

Behaviorally, following anodal stimulation of VMPFC and simultaneous cathodal stimulation of DLPFC (main stimulation group), participants found the presented faces significantly more attractive (*t*(18)=2.26; *P*=0.03; Figure 1c). We tested a number of control conditions in which we varied the location and polarity of tDCS electrodes. None of these control conditions yielded a significant increase in attractiveness ratings following stimulation (Figure 1c, Supplementary Figure S1). Taken together, these control conditions show that the specific combination of electrode placement and anodal/cathodal stimulation in the main stimulation group was critical to cause the behavioral and neural effects reported (F(2, 52)=5.48; *P*=0.007).

For the main group in which anodal stimulation was applied to VMPFC and cathodal stimulation was applied to DLPFC, and the active sham group in which anodal stimulation was applied to DLPFC and cathodal stimulation was applied to VMPFC, we collected fMRI while participants made attractiveness ratings. We made four predictions about the patterns of neural activity resulting from these stimulation conditions, which we tested using the fMRI data. First, in both the stimulation groups, activity in VMPFC should be correlated with participants' attractiveness ratings both before and after stimulation. Second, an interaction between attractiveness ratings before and after stimulation should reveal an increase in neural activity for attractive faces in ventral midbrain following stimulation in the main group as compared with the active sham group (reflecting a remote stimulation of ventral midbrain in the main stimulation group). Third, VMPFC and ventral midbrain should exhibit increased functional connectivity following stimulation in the main group compared with the active sham group. Fourth, those participants with enhanced connectivity between VMPFC and ventral midbrain following stimulation in the main group should display larger increases in attractiveness ratings.

We tested the first prediction by estimating a GLM of BOLD activity that included a parametric regressor for attractiveness ratings at the time of evaluation. Activity in VMPFC was correlated with attractiveness ratings for all participants both before and after stimulation (Figures 2a and b; Supplementary Table S1). The area of VMPFC identified overlaps with regions that have been associated with attractiveness ratings in other studies.^59, 60^

To test the second prediction, we used the same GLM described above. We found significant interactions between attractiveness ratings before and after tDCS in the main group as compared with the active sham group in a ROI, including the ventral midbrain (Figure 2c, Supplementary Table S2; Supplementary Figure S2). This interaction was such that following stimulation in the main group, activity in the ventral midbrain was more positively correlated with attractiveness ratings (Figure 2d). The ventral midbrain has been implicated in responses to rewarding stimuli,^65^ and this interaction suggests that tDCS in the main group increases responsiveness in this region as compared with the active sham group.

**Figure 3:**
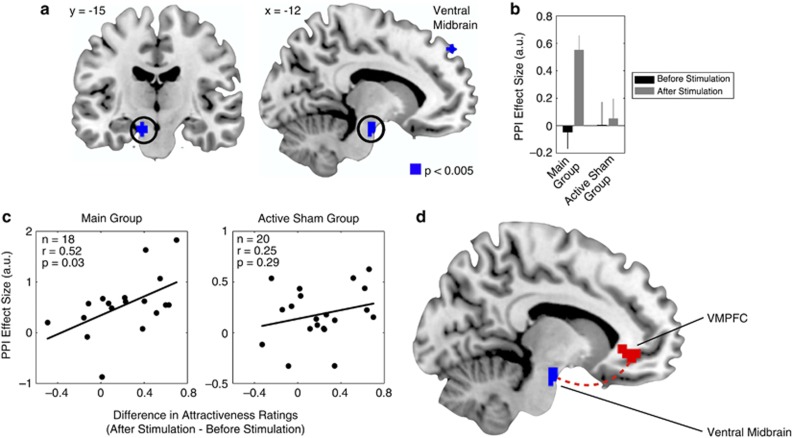
Functional connectivity. (**a**) In the main group as compared with the active sham group, the ventromedial prefrontal cortex (VMPFC) showed positive stimulation-related functional connectivity with a region of ventral midbrain. (**b**) For the psychophysiological interaction (PPI) contrast, average effect sizes representing the functional connectivity between seed activity in the VMPFC and the ventral midbrain. (**c**) In the main group, the more enhanced a participants' functional connectivity between these regions following stimulation, the larger their increase in attractiveness ratings following stimulation. One participant in the main group was removed from this analysis because her PPI parameter estimate constituted a statistical outlier (outside two s.ds. of the mean). (**d**) Diagram summarizing the results of the PPI analyses and illustrating the path through which VMPFC stimulation might enhance activity in the ventral midbrain. All contrasts are displayed at *P*<0.005 uncorrected, and significant at *P*<0.05, small volume corrected. a.u., arbitrary units.

To address our third prediction, we created a new GLM in which we tested a PPI between before/after stimulation (psychological/task variable) and seed activity in the VMPFC (physiological variable). This model allowed us to examine the network effects of VMPFC stimulation on other brain regions, with specific interest in the same ROI used above that encompassed ventral midbrain dopaminergic areas (Supplementary Figure S2). Strikingly, we found a region of the same ventral midbrain ROI to be more correlated with VMPFC activity following stimulation in the main stimulation group as compared with the active sham group (Figures 3a and b; Supplementary Table S3). This result suggests that the functional connectivity between VMPFC and ventral midbrain is enhanced by tDCS in the main stimulation group.

We tested our fourth prediction by performing a linear regression of activities in ventral midbrain identified in the PPI and the differences in participants' mean attractiveness ratings following tDCS. We found a significant correlation in the main stimulation group (*r*=0.52, *P*=0.03) and not the active sham group (*r*=0.25, *P*=0.29). This correlation illustrates that those participants with more enhanced connectivity following tDCS (in the main stimulation group) exhibited the greatest increase in attractiveness ratings (Figure 3c). Thus, anodal stimulation of VMPFC increased the functional connectivity between VMPFC and ventral midbrain (Figure 3d), and the tDCS enhancement of these connections caused participants' increases in behavioral ratings.

## Discussion

These results demonstrate that anodal tDCS of VMPFC and cathodal stimulation of DLPFC can be used to induce remote changes in regions deep within the brain, which were conventionally thought to be unreachable with noninvasive stimulation techniques. Specifically, we were able to elicit remote functional changes within the ventral midbrain, an area populated with SN and VTA neurons and their efferent projections. Moreover, our attractiveness rating results indicate that these tDCS-induced neural changes have a direct influence on participants' rewarding appraisals.

Ours is the first tDCS study that provides a precise neuromechanistically motivated stimulation paradigm, which directly yields both stimulation-induced changes in brain connectivity and corresponding behavioral changes. These results may be related to a recent tDCS study,^25^ which reported that cortical stimulation induced activations in the caudate nucleus. However, that study provided evidence for neither a brain network through which stimulation-induced changes occurred nor offered evidence that such neural changes were directly linked to behavioral effects. Our study goes further by providing simultaneous neural and behavioral evidence consistent with known functions of the remotely stimulated ventral midbrain. Moreover, the neural patterns of functional connectivity we induced with a very specific tDCS electrode configuration (and no other control stimulation conditions) are in concert with the network of projections known to exist between the frontal cortex and ventral midbrain. Indeed, a previous study found that increases in ventral midbrain BOLD are associated with increased reward preferences.^66^

Although fMRI and the paradigm we used in this experiment are not attuned to precisely identify the neural network that gives rise to the tDCS aftereffects we observed, our stimulation paradigm could take advantage of the numerous prefrontal cortex connections to induce the deep-brain changes we observed. The prefrontal cortex has projections that directly interface with the ventral midbrain,^28, 29, 30, 31, 32^ while a far larger number of prefrontal connections indirectly couple the prefrontal cortex and ventral midbrain via the striatum.^5, 33, 34, 35^ We found significantly increased stimulation-induced connectivity between the prefrontal cortex and the ventral midbrain, and our fMRI analysis did not show significant connectivity between the striatum and the prefrontal cortex. A possible explanation for a lack of stimulation-induced connectivity in the striatum, despite its extensive anatomical connections to prefrontal cortex, could be due to a limitation of fMRI. In the context of this experiment, the transmission of signals from the prefrontal cortex through the striatum might be detectable with fMRI only through the striatum's inputs to the ventral midbrain (given that fMRI BOLD signal is more attuned to imaging synaptic processing of afferent input signals as opposed to spiking output^67^). In this view, prefrontal tDCS could induce striatal spiking output, which goes undetected by fMRI and causes the increased activity in the ventral midbrain we observed. Another explanation for the absence of striatal activity in our fMRI analysis is that tDCS of prefrontal cortex could be enhancing the direct prefrontal projections to the ventral midbrain. In both of these explanations, it is possible that what is encoded by the enhanced BOLD signal observed in the ventral midbrain after stimulation is activity within inputs to dopamine neurons.

The gamma-aminobutyric acid (GABA)ergic^68, 69^ and glutamatergic^31, 32^ concentrations in the prefrontal cortex have an important role in modulating activity and dopamine release in the midbrain and striatum, and previous animal^70^ and human studies^71, 72^ have found that anodal and cathodal tDCS influence these neurochemical systems. These studies have found that the aftereffects of anodal tDCS are dependent on modulation of GABA, with anodal tDCS yielding a decrease in GABA concentration at the site of stimulation. Cathodal aftereffects of tDCS, on the other hand, were found to be dependent on the modulation of glutamatergic synapses, yielding decreased concentrations of glutamate following stimulation. Although fMRI does not allow us to directly test such neurochemical effects in the context of our study, the tDCS aftereffects we report could be the result of changes in frontal cortex neurochemistry. Along these lines, in our experiment, VMPFC anodal tDCS could inhibit GABAergic interneurons, which in turn disinhibits pyramidal efferents that project to midbrain dopaminergic neurons, yielding the increase in midbrain sensitivity that we observe in our fMRI results. Cathodal DLPFC stimulation could also contribute to the midbrain activity we observe by reducing cortical glutamatergic concentrations, which in turn disinhibits subcortical dopamine release.

It is important to note that none of the control stimulation conditions yielded significant behavioral or neural effects. This suggests that the singular effects of cathodal or anodal stimulation were not sufficient to yield a significant influence. Instead, the very specific combination of anodal VMPFC and cathodal DLPFC stimulation were required to elicit behavioral and neural effects. However, considerable work will be needed to establish exactly which anatomical and neurochemical pathways are acted upon by this stimulation paradigm, and how interactions between anodal and cathodal stimulation give rise to the neural and behavioral effects we observed.^4^ Since fMRI cannot provide a direct measure of dopaminergic function, future investigation using molecular imaging with dopamine receptor ligands (that is, positron emission tomography) will be needed to directly observe if this tDCS paradigm causes increases in basal ganglia dopamine release. Confirmation of the influence of this tDCS paradigm on dopaminergic activity will open the possibility of its use for the treatment of neuropsychiatric disorders, such as depression and schizophrenia.

Given the ubiquity of the prefrontal cortex and basal ganglia in decision making and motivational performance, it is possible that our stimulation paradigm could influence a wide range of behavioral tasks. Of particular note, decision-making tasks that require higher level of reasoning often recruit DLPFC,^73^ which was the location of our cathodal electrode. With this in mind, future work must take into account how more complicated behavioral tasks might interact with electrode placement and polarity. An overall understanding of how this paradigm interacts with behavioral performance in a variety of tasks will be necessary to evaluate its potential clinical efficacy in patient populations.

In conclusion, we provide an illustration of how a network of interconnected brain areas can be stimulated with tDCS to causally influence deep brain regions containing dopaminergic neurons. We believe that our tDCS protocol is a promising approach to noninvasively modulate midbrain activity and functions that may be disrupted in neuropsychiatric disorders.
