# Supplementary material for: Noninvasive remote activation of the ventral midbrain by transcranial direct current stimulation of prefrontal cortex
Source: Transl Psychiatry. 2013 Jun 11;3(6):e268–. doi: 10.1038/tp.2013.44 (PMC3693403; doi:10.1038/tp.2013.44)
Supplement: Supplementary Information [file tp201344x1.pdf]

SUPPLEMENTARY INFORMATION

Noninvasive Remote Activation of the Ventral Midbrain by Transcranial Direct Current Stimulation of Prefrontal Cortex

Vikram S. Chib[1, 2*], Kyonksik Yun[1, 2*], Hidehiko Takahashi[3,4], Shinsuke Shimojo[1, 2]

[1] Division of Biology, California Institute of Technology
[2] Computation and Neural Systems, California Institute of Technology
[3] Department of Psychiatry, Kyoto University Graduate School of Medicine
[4] Precursory Research for Embryonic Science and Technology (PRESTO), Japan Science and Technology Agency

* Equally contributing authors

To whom correspondence should be addressed: vchib@caltech.edu

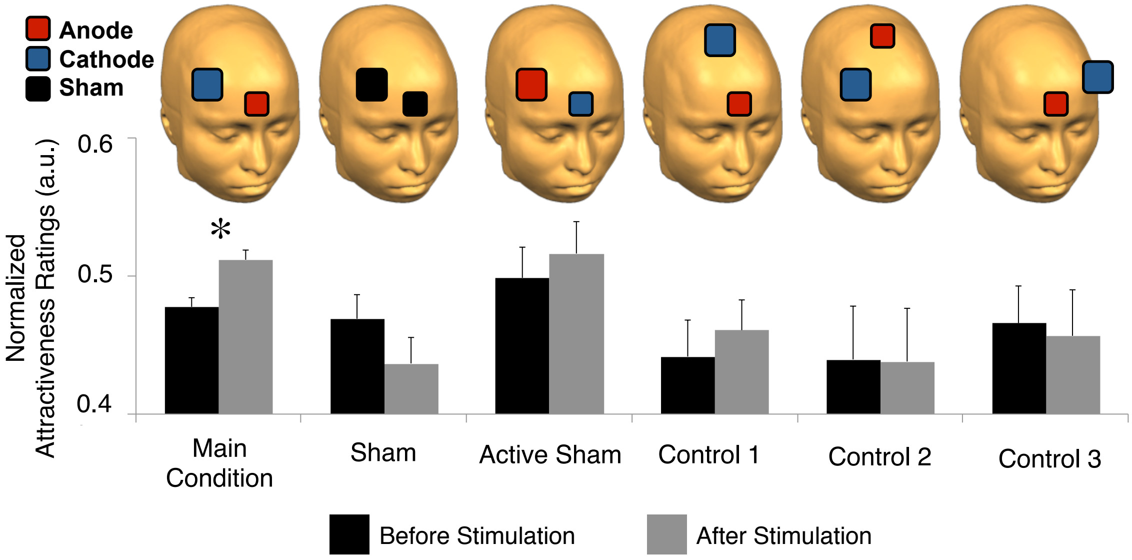

Figure S1. Complete behavioral results. We found a significant interaction between before/after stimulation and stimulation groups (repeated measures ANOVA, $F(5,93)=2.59$, $p=0.03$). We found significant increase of attractiveness ratings in main stimulation condition($t(18)=2.26$, $p=0.037$). No other stimulation conditions resulted in an increase in attractiveness ratings after stimulation.

Ventral Midbrain ROI

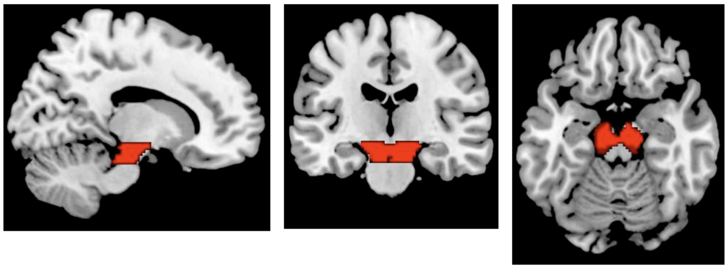

Figure S2. Anatomically defined regions of interest (ROI) for the ventral midbrain (encompassing the VTA and SN).

Significant Regions in Imaging Analyses

For the contrasts reported in the main text, we report results in a priori regions of interest previously identified in neuroimaging studies on decision-making and reward (ventral midbrain, striatum, dorsolateral prefrontal cortex, ventromedial prefrontal cortex). For completeness peaks are reported for all clusters ≥ 10 at $p < 0.005$. Statistically significant activations are those found in apriori regions of interest FDR $p<0.05$, and those regions that survive whole-brain correction for multiple comparisons at $P < 0.05$ (indicated with *).

Table S1. A conjunction analysis between regions showing a signal positively correlated with attractiveness ratings before and after stimulation in the main stimulation and the active sham groups.

Region	Laterality	x	y	z	z-score
Motor Cortex	L	-33	-33	57	7.70
Cerebellum	R	21	-51	-24	4.91
Primary Auditory Cortex	L	-54	-15	6	3.50
Ventromedial Prefrontal Cortex	L	-9	39	-6	3.42*
Anterior Cingulate	C	0	33	6	3.36

Table S2. Regions showing a interaction between attractiveness ratings before and after tDCS in the main group as compared to the active sham group.

Region	Laterality	x	y	z	z-score
Ventral Midbrain	C	0	-15	-15	3.64*

Table S3. Regions showing increased stimulation related functional connectivity with the VMPFC in the main stimulation group compared to the active sham group.

Region	Laterality	x	y	z	z-score
Ventral Midbrain	L	-12	-15	-21	4.23*
Frontal Eye Fields	R	30	48	42	3.54
Frontal Eye Fields	L	-15	51	45	3.46
Orbitofronal Cortex	L	-21	30	-15	3.33
